# Multiscale Synergistic Investigation on the Mechanical and Tribological Performances of Graphene-Reinforced PEEK/PTFE Composites

**DOI:** 10.3390/polym18030308

**Published:** 2026-01-23

**Authors:** Yan Wang, Kaiqi Dong, Henan Tang, Bin Yang, Shijie Wang

**Affiliations:** School of Mechanical Engineering, Shenyang University of Technology, Shenyang 110870, China

**Keywords:** polytetrafluoroethylene, polyetheretherketone, graphene, molecular dynamics, experimentation

## Abstract

Polytetrafluoroethylene (PTFE) is a self-lubricating material but has poor wear resistance. The wear resistance of the composites was enhanced by the incorporation of polyetheretherketone (PEEK), whereas the friction-reducing performance was compromised, thus resulting in an inherent trade-off between wear resistance and lubricity. Graphene nanosheets (GNSs) with high strength and lubricity were introduced as a reinforcement for PEEK/PTFE composites. Composite specimens with varying GNS contents were fabricated and characterized for their mechanical and tribological properties and wear morphologies. Combined with molecular dynamics (MD) simulations, the micro-mechanisms were further elucidated. The optimal GNS content was determined to be 2 wt%, which improved the tensile strength by 10.58% and reduced the wear rate by 17.88% compared to PEEK/PTFE. It achieved the synchronous enhancement of mechanical strength and wear resistance while maintaining desirable friction-reducing performance. MD simulation results demonstrated that the strong interfacial interactions between GNSx and the polymer enabled GNSs to adsorb polymer chains and form a dense rigid network with reduced free volume (FV). The mechanical properties were enhanced by efficient load transfer and the suppression of interfacial delamination enabled by this unique structure; meanwhile, wear resistance was improved due to the mitigation of friction-induced molecular chain scission.

## 1. Introduction

Polytetrafluoroethylene (PTFE) is a high-performance engineering plastic widely employed in critical applications including aerospace seals, petrochemical pipelines, and automotive mechanical bearings [1,2,3]. Its extensive application can be attributed to its exceptional chemical resistance, oxidation stability, and intrinsic self-lubricating properties. However, the unique molecular structure of PTFE renders it inherently deficient in mechanical performance, characterized by insufficient load-bearing capacity, a low elastic modulus, and poor wear resistance [4]. These limitations significantly restrict its application under high-load and high-wear conditions. Extensive investigations have been conducted to enhance the mechanical properties and hardness of PTFE composites. Various fibers, metal particles, and special engineering plastics are commonly adopted as functional fillers for PTFE. Rahul et al. [5] studied the tribological properties of basalt fiber-reinforced polyimide and polytetrafluoroethylene composites under various wear conditions. Yang et al. [6] demonstrated that the incorporation of aluminum oxide could significantly reduce the wear loss of PTFE coatings. Andrey et al. [7] enhanced the mechanical and tribological properties of polytetrafluoroethylene using composite fillers comprising carbon fibers, zirconia, silica, and boron nitride.

Polyetheretherketone (PEEK) is a semicrystalline thermoplastic high-performance engineering plastic endowed with excellent corrosion resistance, fatigue resistance, lightweight characteristics, and cost-effectiveness. It is widely employed in harsh mechanical friction conditions and the aerospace field [8]. PEEK is often adopted as the matrix phase in PTFE-reinforced composites owing to its well-balanced properties. Studies have confirmed that an optimal balance between wear resistance and mechanical properties is achieved for the composites with 10 wt% PEEK [9,10]. Nevertheless, this modification compromises the inherent superior friction-reducing performance of PTFE, restricting its use in lubrication-sensitive applications such as high-precision seals and low-friction bearings. This consequently results in an intractable trade-off between wear resistance and lubricity [11].

Graphene nanosheets (GNSs) with ultra-high tensile strength, high elastic modulus, and superior lubricating characteristics derived from their atomically smooth sp^2^ structure were innovatively introduced as hybrid fillers to resolve this challenge. The synergistic interaction between GNSs and the PEEK/PTFE matrix is expected to break the existing performance bottleneck of the composite system. Conventional modified fillers such as molybdenum disulfide, carbon fibers suffer from inherent drawbacks including large particle sizes and poor interfacial compatibility [12,13,14]. By contrast, carbon nanomaterials exhibit distinctive advantages in polymer modification, which are attributed to their prominent nanoscale effects and high specific surface area. Arash et al. [15] demonstrated that increasing the aspect ratio of carbon nanotubes can significantly enhance interfacial bonding and the overall modulus of composites. Chen et al. [16] studied amino-modified carbon nanotube/polyimide composite films and successfully fabricated flexible composite films with high dielectric constants and ultralow dielectric loss. Zhu [17] found that the addition of 5% graphene significantly enhanced the tribological properties of the material. Chawla et al. [18] investigated the properties of styrene butadiene rubber/graphene oxide composites and demonstrated enhancements in both mechanical and tribological performance. Patil et al. [19] prepared nickel-based nanocomposites reinforced with GNSs, which exhibited significantly improved mechanical properties compared with pure nickel, including a yield strength approximately four times higher. Larsson et al. [20] concluded that GNS reinforced epoxy composites exhibit an increase in stiffness of 10–30% relative to the neat epoxy matrix. Previous studies have shown that a small amount of GNSs can substantially enhance the mechanical properties and wear resistance of polymers while preserving their intrinsic characteristics [21,22,23,24].

However, most existing research has been primarily confined to macroscopic experimental analysis, with a predominant focus on performance characterization and phenomenological description. Studies concerning the micromechanisms that underpin the reinforcing effect of GNSs in complex multicomponent systems remain relatively scarce. Thus, systematic investigations are urgently required to elucidate the intrinsic action mechanisms of GNSs within such composite systems.

Molecular dynamics (MD) simulation can effectively elucidate the interfacial interaction mechanisms, energy evolution laws, and molecular motion behaviors within materials as an atomic-scale computational approach [25,26]. Cui et al. [27] studied the mechanical and tribological properties of nitrile rubber reinforced with differently functionalized graphene sheets via MD simulations. Qiao et al. [28] investigated the wear properties of a Fe_2.5_Ni_2.5_CrAl multi-principal element alloy using combined experimental and MD simulation methods. Samanta et al. [29] revealed that 2 wt% GNSs can increase the yield strength of polyvinyl alcohol composites from 611 MPa to 636 MPa via MD simulations. Li et al. [30] indicated that GNSs can enhance the Young’s modulus, shear modulus, and hardness of the polymer matrix by 150%, 27.6%, and 35%, respectively. Juma et al. [31] pointed out that a GNS with its intact sp^2^ structure can improve the tensile strength of polypropylene to 160.35 MPa. Yang et al. [32] demonstrated that functionalized GNSs can enhance the mechanical properties of PTFE composites by strengthening interfacial bonding, albeit leading to an increase in the friction coefficient. Shiu et al. [33] confirmed that GNS can increase the Young’s modulus of epoxy resin to 5.48 GPa and elevate the glass transition temperature to 390 K, clarifying the micro-mechanism of nano-reinforcement at the atomic scale.

Building upon the aforementioned research, this study adopts a combined methodology of MD simulations and experimental investigations to systematically explore the reinforcement mechanism of GNSs in PEEK/PTFE composites, as well as its influence on the microstructure. Composite specimens of GNS/PEEK/PTFE with different proportions were fabricated, followed by mechanical and tribological performance tests. The reinforcement mechanisms of GNSs on the mechanical and tribological properties of the composites are elucidated by simulating and analyzing parameters of the composite system, including free volumes (FV) distribution, total potential energy, and interfacial interaction energy. This research provides a solid theoretical foundation and novel research insights for the investigation and practical application of PTFE-based composites. It also facilitates the further optimization of composite performance and the expansion of their potential application scopes.

## 2. Experiments

### 2.1. Raw Materials and Pretreatment

PTFE was supplied by Daikin Fluorochemicals Co., Ltd. (Kashima, Japan), with an average particle size of 40 μm and a density of 2.14 to 2.20 g/cm^3^. PEEK was provided by Jilin Zhongyan Polymer Materials Co., Ltd. (Changchun, China), exhibiting an average particle size of 24 μm and a density of 1.4 g/cm^3^. GNS powder, characterized by a multilayer structure with 5 to 10 layers and a thickness of 3.4 to 8 nm, and an average flake diameter of 25 μm, was obtained from Suzhou Carbon Feng GNS Technology Co., Ltd. (Suzhou, China). To prevent any potential influence on subsequent experimental results, the purity of all raw materials was verified by XRD diffraction analysis prior to use.

### 2.2. Preparation of Composite Samples

First, PTFE and PEEK powders were placed in a vacuum oven (Model: DZF-6055, Shanghai Yiheng Scientific Instruments Co., Ltd., Shanghai, China) at 150 °C and dried for 3 h to remove moisture adsorbed on the powder surfaces. They were then immediately sealed and stored to prevent moisture reabsorption. According to the proportions listed in Table 1, PTFE, PEEK, and GNS powders were weighed using a 0.1 mg precision electronic balance. The blended raw materials were transferred to a high-speed mixer (Model: SHR-10, Changzhou Haizheng Pharmaceutical & Chemical Equipment Co., Ltd. Changzhou, China) and mixed at 1200 r/min for 60 s per cycle. The mixing process was repeated 10–15 times to ensure uniform dispersion [34]. After mixing, the materials were cooled to room temperature and sealed to avoid contamination by dust.

Subsequently, the mixed powders were cold-pressed into shape using a hydraulic molding machine. The process was carried out under a molding pressure of 30 MPa [35] and a pressing speed of 20 mm/min, with the pressure maintained for 30 min to ensure compactness of the green body. The cold-pressed compact was then allowed to stand for over 12 h to relieve internal stresses through natural aging, thereby preventing sample cracking caused by stress concentration during subsequent heating. Finally, the demolded green body was placed in a programmable heating furnace, heated from room temperature to 360 °C at a rate of 50 °C/h, held for 3–4 h to achieve complete sintering, and then slowly cooled to room temperature at the same rate. The entire sintering process was conducted in an air atmosphere. The sintered sample was machined on a CNC lathe to meet the testing standards.

### 2.3. Experimental Testing and Characterization

#### 2.3.1. Mechanical Properties Testing

Tensile and compression tests were performed using an AUTOGRAPH AG-X series vertical electronic universal testing machine (Shimadzu, Kyoto, Japan). Tensile tests followed the standard “Determination of tensile properties of plastics,” [36], and the compression tests were conducted according to the standard “Determination of compressive properties of plastics,” [37]. For the tensile tests, the specimens had a diameter of 6 mm (±0.4 mm) in the reduced section of the gauge length and a gauge length of 33 mm (±2 mm), and the overall length of the tensile specimens was 80 mm. The specimens for compression tests were prepared with dimensions of 10 mm × 10 mm × 4 mm. The cross-head speed was set to 50 mm/min for the tensile tests and 2 mm/min for the compression tests. The maximum load and the extension at fracture were recorded to calculate the tensile strength (*σ* = *F*_b_/*S*_o_, where *F*_b_ is the maximum load and *S*_o_ is the original cross-sectional area of the specimen) and tensile elongation (*e* = [(*L*_k_ − *L*_0_)/*L*_0_] × 100%, where *L*_k_ is the gauge length after fracture and *L*_0_ is the original gauge length). The compressive deformation was calculated as *c* = [(*T*_0_ − *T*_i_)/(*T*_0_ − *T*_n_)] × 100%, where *T*_n_ is the thickness of the spacer, *T*_0_ is the specimen thickness before testing, and *T*_i_ is the specimen thickness after testing. In this study, the value of *T*_n_ was set to 0 since no spacers were used during the compression tests. Each test was repeated three times, and all mechanical testing data are presented as the average values of each group to enhance the reliability and minimize random errors.

#### 2.3.2. Tribological Properties Testing

Friction and wear tests were performed on a THORLABS corrosion fretting wear testing machine (Newton, NJ, USA). A ball-on-flat configuration was employed, using QT450 spheres as the counterface in reciprocating dry sliding tests without lubrication. The test parameters were set as follows: The friction specimen size was 20 mm × 10 mm × 5 mm. load 200 N, frequency 2 Hz, room temperature, stroke length 10 mm, sliding speed 0.04 m/s, and test duration 30 min. After testing, the mass of each specimen before and after wear was measured using an electronic balance with a precision of 0.1 mg to calculate the wear rate. The wear rate was calculated using Equation (1).

(1) $$W=\frac{△m}{\rho \cdot F_{n}\cdot L}$$

where *W* denotes the specific wear rate, ∆m is the mass loss of the specimen, ρ represents the density of the composite, *F*_n_ is the normal load, and *L* is the sliding distance. The friction coefficient was recorded throughout the test by the equipment’s software. Specifically, three sets of parallel experiments were completed in the test, and the average value of the three experiments was taken as the final data.

#### 2.3.3. Worn Surface Morphology and Composition Characterization

The worn surface morphology of the samples was examined using a Zeiss Sigma 300 field emission scanning electron microscope (SEM) (Zeiss, Oberkochen, Germany) to analyze the wear mechanisms. Prior to imaging, the surfaces were sputter-coated with a thin gold layer (approximately 5 nm thick) to enhance conductivity. The SEM operating conditions included an accelerating voltage of 15 kV and magnifications ranging from 300× to 5000×. In addition, X-ray photoelectron spectroscopy (XPS) was employed to analyze the chemical composition of the sample surfaces. The analysis focused on changes in the binding energies of C, F, and O elements to investigate the interfacial interactions between GNSs and the polymer matrix.

### 2.4. Molecular Dynamics Simulation Details

#### 2.4.1. Construction and Optimization of Amorphous Models

**Figure 1:**
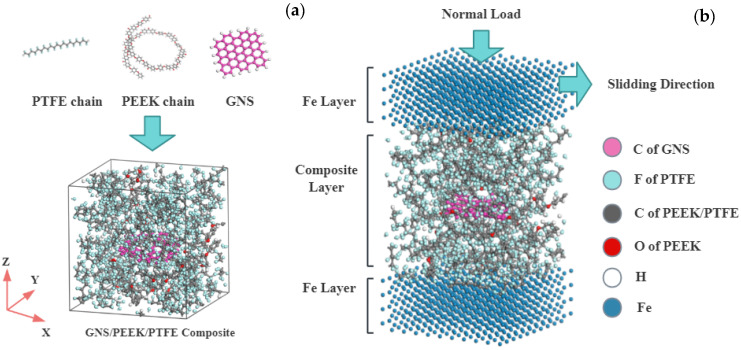
Molecular model of GNS/PEEK/PTFE composite: (**a**) GNS/PEEK/PTFE composite model, (**b**) GNS/PEEK/PTFE composite friction model.

MD simulations were performed using Materials Studio 2018 software to construct amorphous models of GNS/PEEK/PTFE composites with varying GNS contents (1%, 2%, and 3%), as illustrated in Figure 1a.

Since the modeling methods for composites with different GNS contents are consistent, only the model with 2 wt% GNS content is taken as an example to illustrate the specific modeling process in this study. First, a periodic cubic lattice measuring 3 × 3 × 3 nm^3^ was created [38], and a single-layer GNS (2.9 × 2.9 nm^2^) was placed at its center. Hydrogen atoms were attached to the GNS edges to eliminate boundary effects. PTFE and PEEK molecular chains, each containing 10 repeating units, were built and randomly packed into the lattice at a density of 2.0 g/cm^3^ using the Monte Carlo [39] method with a random number algorithm, forming the initial amorphous model as shown in Figure 1a. The resulting lattice system consisted of 1 PEEK molecular chain, 26 PTFE molecular chains, and 1 piece of GNS, with the mass fractions of GNS, PEEK, and PTFE consistent with the experimental formulation.

The initial model underwent geometric optimization and dynamic relaxation. Geometric optimization was conducted using a combination of the steepest descent and conjugate gradient methods, with energy and force convergence thresholds set to 1 × 10^−5^ kcal/mol and 1 × 10^−2^ kcal/(mol·nm), respectively, to ensure the system reached a local energy minimum. Dynamic relaxation was carried out in two stages: first, NVT ensemble equilibration at 298 K with an Andersen thermostat [40] for 0.5 ns with a 1 fs time step to release internal stresses; second, a 1 ns NPT ensemble molecular dynamics simulation was performed under the conditions of 298 K and 101 kPa using a Berendsen barostat [41], and the final equilibrium density of the system was determined to be 1.95 g/cm^3^. The system was deemed to have reached a stable equilibrium state when the fluctuation ranges of temperature, pressure, and potential energy were within 5% after equilibration. All simulations employed the COMPASS force field. Van der Waals interactions were computed using an atom-based summation method, and electrostatic interactions were treated with the Ewald method, using a cutoff distance of 1.25 nm [42,43,44].

#### 2.4.2. Simulation of Mechanical Properties

**Figure 2:**
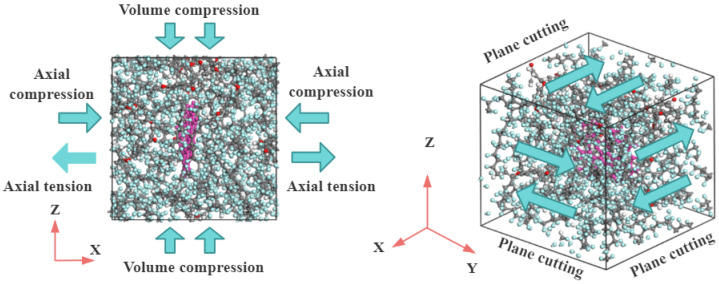
Schematic diagram of constant strain method for mechanical property simulation.

The lowest-energy configuration obtained from the NPT dynamics equilibration simulation described in the previous section was selected. Taking the last frame of this configuration as the initial state, a further 200 ps NVT MD simulation was performed, with all simulation parameters kept consistent with those used in the initial NVT dynamics stage. Mechanical properties were evaluated via the constant strain method by applying four strain levels (*ε* = −0.003, −0.001, 0.001, 0.003) along six independent directions (x, y, z, xy, xz, yz) of the composite system, with a maximum strain amplitude of 0.003. For the dynamically optimized model, the lowest energy configuration determined from the simulated annealing was selected, and a 200 ps NVT equilibration was performed, with all other parameters kept the same as in the initial NVT equilibration. To ensure the reliability of the simulation results, all the above-mentioned simulations related to mechanical properties were independently performed 3 times based on the model after NVT equilibration, and the final mechanical property data were taken as the arithmetic mean of the three simulation results. The configuration is presented in Figure 2.

Based on the definition of virial stress, stress components were computed in each direction. Young’s modulus was derived using the relations *E*_i_ = *σ*_1_/*ε*_1_, *σ*_i_ = *C*_ij_*ε*_j_, and *ε*_i_ = *S*_ij_*σ*_j_, where *σ*_i_ represents stress, *ε*_i_ represents strain, *C*_ij_ is the stiffness matrix, and *S*_ij_ is the compliance matrix.

The bulk modulus (*B*) and shear modulus (*G*) of the composite system were calculated using Equations (2)–(5). Following the Voigt–Reuss–Hill averaging scheme, the effective bulk modulus (*B*_H_) and shear modulus (*G*_H_) were determined from Equations (6) and (7).

(2) $$B_{V}=\frac{1}{9}\left(\sum_{i=1}^{3}C_{\;\mathrm{ii}}+2\sum_{\begin{array}{l} i=1\\ i<j \end{array}}^{3}C_{\;\mathrm{ij}}\right)$$

where *C*_ij_ denotes the stiffness matrix, where the subscripts i and j range from 1 to 6, representing the six independent stress and strain components (specifically, 1, 2, 3 correspond to normal stresses and strains along the x, y, z directions, while 4, 5, 6 correspond to shear stresses and strains).

(3) $$G_{V}=\frac{1}{15}\left[\left(\sum_{i=1}^{3}C_{\mathrm{ii}}-\sum_{\begin{array}{l} i,\;j=1\\ i<j \end{array}}^{3}C_{\mathrm{ij}}\right)+3\sum_{i=4}^{6}C_{\mathrm{ii}}\right]$$

where *S*_ij_ is the compliance matrix, which is the inverse of the stiffness matrix and describes the ease with which a material deforms under stress.

(4) $$B_{R}={\left(\sum_{i=1}^{3}S_{\mathrm{ii}}+2\sum_{\begin{array}{l} i,\;j=1\\ i<j \end{array}}^{3}S_{\mathrm{ij}}\right)}^{-1}$$

where *B*_R_ is the Reuss bulk modulus. It is calculated based on the assumption of stress uniformity and typically represents the lower bound of the equivalent modulus.

(5) $$G_{R}=\frac{15}{4\left(\sum_{i=1}^{3}S_{\mathrm{ii}}-\sum_{\begin{array}{l} i,\;j=1\\ i<j \end{array}}^{3}S_{\mathrm{ij}}\right)+3\sum_{i=4}^{6}S_{\mathrm{ii}}}$$

where *G*_R_ is the Reuss shear modulus. It is calculated based on the assumption of stress uniformity and represents the lower bound of the equivalent shear modulus.

(6) $$B_{H}=\frac{1}{2}\left(B_{V}+B_{R}\right)$$

where *B*_H_ represents the equivalent bulk modulus, defined as the arithmetic average of the Voigt and Reuss bounds according to the VRH.

(7) $$G_{H}=\frac{1}{2}\left(G_{V}+G_{R}\right)$$

where *G*_H_ represents the effective shear modulus, defined as the arithmetic average of the Voigt and Reuss bounds according to the VRH. Three parallel calculations were carried out during the simulation process, and the average value of the three calculations was taken as the final data.

#### 2.4.3. Simulation of Tribological Properties

A three-layer friction model was constructed, as shown in Figure 1b. The model consists of Fe atomic layers (2.9 × 2.9 × 1.1 nm^3^) at the top and bottom, with a middle layer of PEEK/PTFE composite containing varying GNS contents. Initially, with the Fe layers fixed, five cycles of high-low temperature annealing (300–500 K, in 50 K increments) were performed to allow full relaxation of the composite material. Subsequently, the positional constraints on the Fe layers were removed, a sliding velocity of 0.01 nm/ps was applied to the top Fe atoms, and a 500 ps simulation was carried out under the NVT ensemble at 298 K. Trajectory files during the friction process were recorded, and the friction coefficient (*μ* = *F*_f_/*F*_n_, where *F*_f_ is the friction force and *F*_n_ is the normal force) and wear rate (AR = *N*_leave_/*N*_total_, where *N*_leave_ is the number of atoms detached from the substrate and *N*_total_ is the total number of atoms in the original substrate) were calculated. Three parallel calculations were performed for the simulation, and the average values were taken as the final results.

#### 2.4.4. Simulation of GNS Pull-Out Behavior

**Figure 3:**
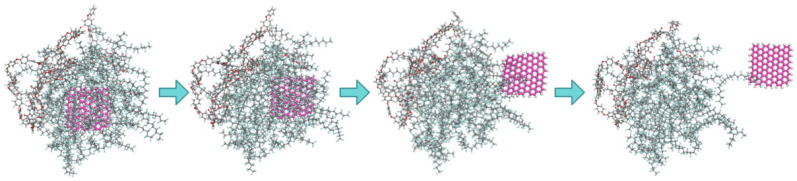
Snapshots of GNS pull-out process in 2%GNS/10%PEEK/PTFE composite.

To investigate the interface reinforcement mechanism, a GNS pull-out simulation was conducted using a 2 wt% GNS model as an example: the GNS was pulled out from the composite system at a speed of 0.1 nm/ps, and the pull-out trajectory snapshots are shown in Figure 3. During the pull-out process, the interfacial interaction energy and the matrix temperature changes were recorded to analyze the bonding strength between GNSs and the matrix.

## 3. Results and Discussion

### 3.1. Mechanical Properties of Composites

**Figure 4:**
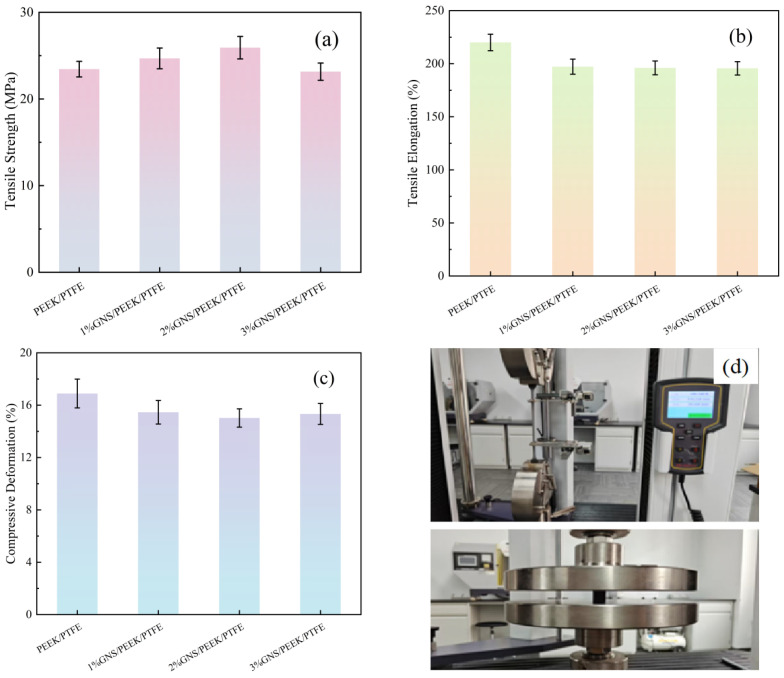
Experimental mechanical properties of GNS/PEEK/PTFE composites: (**a**) tensile strength, (**b**) tensile elongation, (**c**) compressive deformation and (**d**) tensile and compressive processes.

The mechanical properties of the composites were evaluated through compression and tensile tests. The measured mechanical properties, including tensile strength, tensile elongation, compressive deformation, as well as the tensile and compressive processes, are summarized in Figure 4.

According to the data in Figure 4a, the tensile strengths of the 1%GNS/PEEK/PTFE and 2%GNS/PEEK/PTFE composites were 24.68 MPa and 25.92 MPa, respectively. Compared with the PEEK/PTFE matrix (23.44 MPa), these values showed increases of 5.29% and 10.58%. In contrast, the tensile strength of the 3% GNS/PEEK/PTFE composite was 23.15 MPa, which was 1.23% lower than that of the PEEK/PTFE matrix. Based on the tensile elongation data in Figure 4b, the matrix without GNS incorporation exhibited the maximum tensile elongation of 220.03%. With an increase in GNS content, this value gradually decreased to 195.62%. The calculated compressive deformation values for GNS/PEEK/PTFE composites with different compositions are listed in Figure 4c. As shown in Figure 4c, the compressive deformation of all GNS-reinforced composites was lower than that of the matrix (16.89%). The 2 wt% GNS composite demonstrated the optimal performance, exhibiting a compressive deformation of only 15.02%, which represents an 11.07% reduction compared to the matrix. The 1 wt% and 3 wt% GNS composites showed compressive deformations of 15.46% and 15.33%, respectively. Although these values indicated a definite improvement over the PEEK/PTFE matrix, the performance of these two composites was inferior to that of the 2 wt% GNS formulation. The tensile and compressive testing procedures corresponding to the four composite systems are visually illustrated in Figure 4d.

The aforementioned trends in mechanical properties can be ascribed to the intrinsic rigidity of GNS and its dispersion behavior within the polymer matrix. At low GNS loadings (1 wt% and 2 wt%), GNS nanoparticles can be uniformly dispersed in the matrix. For one thing, the GNS transfers tensile stresses via interfacial interactions, thereby enhancing the tensile strength of the composites. For another, it constructs a rigid supporting network inside the matrix, which effectively distributes compressive loads and reduces plastic deformation. Simultaneously, this rigid network enhances the overall stiffness of the material, which in turn induces a decrease in tensile elongation. Among the investigated formulations, the 2 wt% GNS loading achieves an optimal balance between dispersion uniformity and supporting efficiency, thus yielding the most favorable comprehensive mechanical properties. However, when the GNS loading exceeds 2 wt%, strong interlayer van der Waals forces trigger GNS agglomeration. This agglomeration not only impedes stress transfer during the tensile process but also generates internal structural defects. Consequently, both tensile strength and compressive performance declined. In addition, it results in an inhomogeneous supporting structure during the compression process. Consequently, both the tensile strength and compressive performance of the composite undergo a decline. These findings further corroborate that 2 wt% is the optimal GNS loading, which significantly improves the adaptability of the composite for heavy-load service applications.

### 3.2. Tribological Properties of Composites

#### 3.2.1. Friction Coefficient and Wear Rate

**Figure 5:**
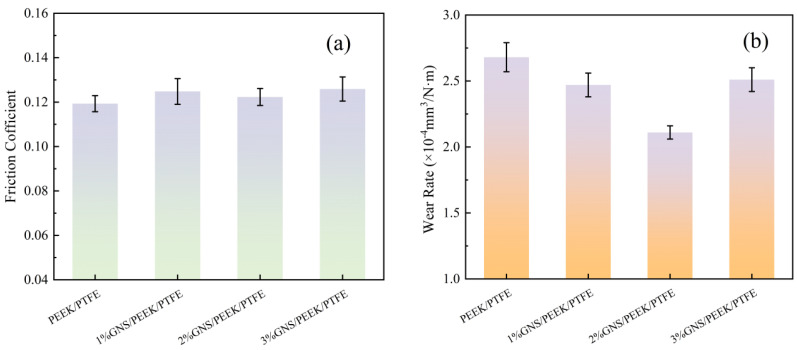
COF and wear rate of GNS/PEEK/PTFE composites: (**a**) friction coefficient, (**b**) wear rate.

The friction coefficients and wear rates of the PEEK/PTFE matrix, as well as the composites modified with 1 wt%, 2 wt%, and 3 wt% GNS, are presented in Figure 5a and Figure 5b, respectively. In terms of friction performance, compared with the matrix (0.1193), the friction coefficients of the 1 wt%, 2 wt%, and 3 wt% GNS composites increased by 4.61%, 2.51%, and 5.53% respectively. The composite with 2 wt% GNS loading exhibits the smallest increment in friction coefficient. In terms of wear resistance, the wear rates of the three composite groups decrease by 5.6%, 17.88%, and 6.2%, respectively, relative to the matrix, with the 2 wt% GNS composite yields the most pronounced reduction.

The aforementioned phenomena demonstrate that the GNS loading content exerts a direct regulatory effect on the friction and wear behaviors of the composites. When the GNS content is 2 wt%, it can be uniformly dispersed in the system. This uniform not only provides rigid support to reduce matrix adhesion but also alleviates frictional resistance through interlayer sliding. As a result, the increase in the friction coefficient is minimized. Simultaneously, it forms a continuous, intact protective barrier at the friction interface, which can effectively withstand the applied load and mitigate direct abrasive wear of the matrix. This robust interfacial barrier consequently endows the composite with optimal overall tribological performance. In contrast, an insufficient GNS loading (1 wt%) results in inadequate interfacial support and the formation of a discontinuous protective barrier. On the other hand, an excessive GNS loading (3 wt%) triggers severe GNS agglomeration. This agglomeration not only intensifies the fluctuation of frictional resistance but also compromises the structural integrity of the protective barrier. Both scenarios consequently lead to suboptimal friction and wear performance of the composites.

In summary, the incorporation of GNSs enhances the tribological performance of the composites by improving the structural stability and deformation resistance of the polymer matrix. Notably, the composite with 2 wt% GNS loading achieves an optimal balance between a low friction coefficient and a low wear rate.

#### 3.2.2. Worn Surface Morphology Analysis

**Figure 6:**
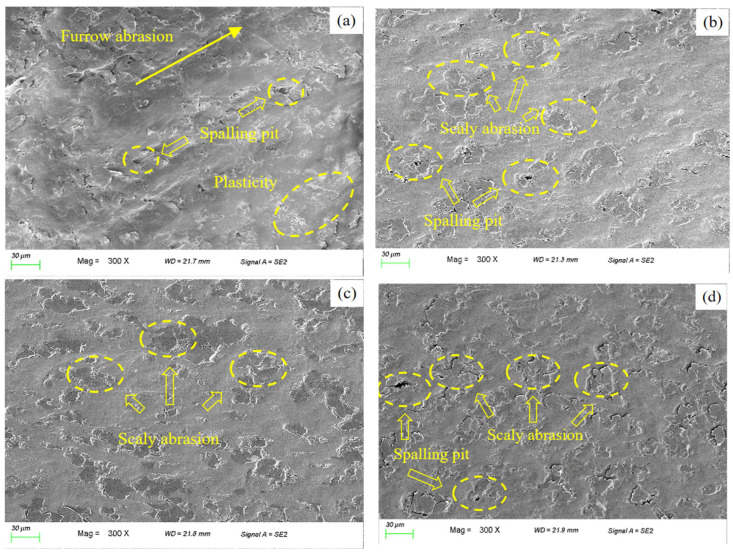
Worn surface morphology of GNS/PEEK/PTFE composites observed by SEM: (**a**) PEEK/PTFE, (**b**) 1%GNS/PEEK/PTFE, (**c**) 2%GNS/PEEK/PTFE, (**d**) 3%GNS/PEEK/PTFE.

To investigate how the GNS influences the tribological properties of PEEK/PTFE composites, we examined the worn surfaces of four specimen groups with a Zeiss Sigma 300 field emission scanning electron microscope as shown in Figure 6.

As illustrated in Figure 6a, the PEEK/PTFE composite exhibited a pronounced wear phenomenon. The wear was characterized by distinct ploughing grooves, numerous spallation pits, significant plastic deformation, and abundant flake-like debris. These features indicate that adhesive wear is the primary mechanism, accompanied by secondary abrasive wear. Under sliding contact, PTFE molecular chains underwent shear-induced scission. The resulting wear debris adhered to the counterface. Then, it ploughed the composite surface, accelerating material removal.

As shown in Figure 6b–d, the GNS-reinforced composites exhibit substantially smoother worn surfaces with shallower wear traces compared to the unreinforced matrix. With a 1 wt% GNS content, microstructural examination shows a considerable reduction in spallation pits, with only minor flake-like features remaining. At this concentration, the GNS formed dispersed support points that reduced matrix adhesion. As a result, the wear mechanism changes to mild adhesive wear. However, the insufficient GNS quantity still permitted limited debris generation. With a 2 wt% GNS content, spallation pits were nearly eliminated, leaving only minimal flake-like marks. This suggests uniform GNS dispersion within the composite with negligible agglomeration. The uniformly dispersed GNS effectively withstands the applied stresses and preserves the structural integrity of the matrix, resulting in a mild adhesive-abrasive wear mode that corresponds well with the low macroscopic wear rate of the composite. At a GNS loading of 3 wt%, spallation pits reappeared alongside increased flake-like debris. This deterioration results from GNS agglomeration, which compromised the local interfacial bonding strength between the filler and matrix. During friction, these agglomerates detached and formed abrasive particles that scratched the matrix surface, reestablishing adhesive wear as the dominant mechanism with secondary abrasive wear. This microstructural evolution further accounts for the limited improvement in the macroscopic wear rate of the composite.

### 3.3. Mechanical Properties of Composites: MD Simulation Results and the Enhancement Mechanism of GNS

**Figure 7:**
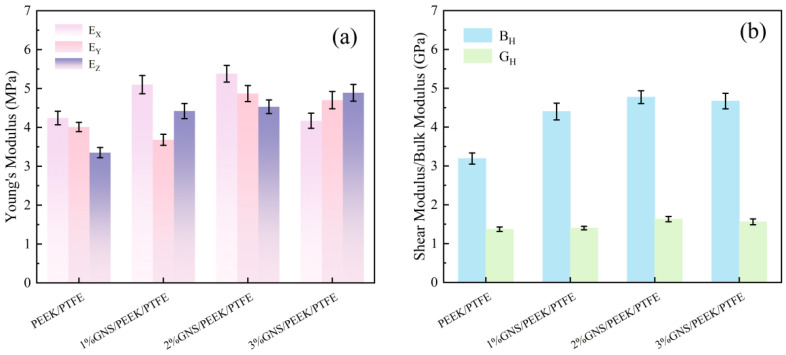
Simulated mechanical properties of GNS/PEEK/PTFE composites (GPa): (**a**) Young’s modulus, (**b**) shear modulus and bulk modulus.

The actual bulk modulus (*B*_H_) and shear modulus (*G*_H_) of the composite system were determined via MD simulations, and the corresponding results are summarized in Figure 7.

The symbols *E*_X_, *E*_Y_, and *E*_Z_ denote the Young’s moduli along the x, y, and z directions, respectively; *E*_MAX_ indicates the maximum Young’s modulus; *B*_R_ and *B*_V_ represent the Reuss and Voigt bulk moduli, respectively; and *G*_R_ and *G*_V_ refer to the Reuss and Voigt shear moduli, respectively.

As shown in Figure 7a, the incorporation of GNSs significantly enhanced the Young’s modulus, bulk modulus, and shear modulus of the PEEK/PTFE matrix. Specifically, the Young’s modulus of the composites with 1 wt%, 2 wt%, and 3 wt% GNS loading were determined to be 5.1 GPa, 5.38 GPa, and 4.89 GPa, respectively. These values represent increases of 20.28%, 26.89%, and 15.53%, respectively, relative to the Young’s modulus of the PEEK/PTFE matrix (4.24 GPa). These results confirm the effectiveness of GNS in restricting molecular chain deformation. As shown in Figure 7b, the bulk modulus of the composites with 1 wt%, 2 wt%, and 3 wt% GNS loading were determined to be 4.4 GPa, 4.77 GPa, and 4.67 GPa, respectively. These values correspond to increases of 37.93%, 49.53%, and 46.39% relative to the PEEK/PTFE matrix (3.19 GPa). This trend is consistent with the minimal macroscopic compressive deformation observed in the experimental tests. Furthermore, the shear modulus of the composites with 1 wt%, 2 wt%, and 3 wt% GNS loading were measured as 1.4 GPa, 1.63 GPa, and 1.56 GPa, respectively. In comparison with the PEEK/PTFE matrix (1.37 GPa), the shear modulus of the composites increased by 2.19%, 18.98%, and 13.87%. These results further confirm that the incorporation of GNS can effectively mitigate shear-induced damage during the friction process. Atomic-scale simulation results demonstrate that the GNS reinforces the constraint of molecular chains and strengthens interfacial interactions. These effects consequently enhance the stiffness, resistance to volumetric deformation, and shear strength. The most pronounced reinforcing effect is observed at a GNS loading of 2 wt%. This observation is strongly corroborated by the corresponding macroscopic experimental data.

**Figure 8:**
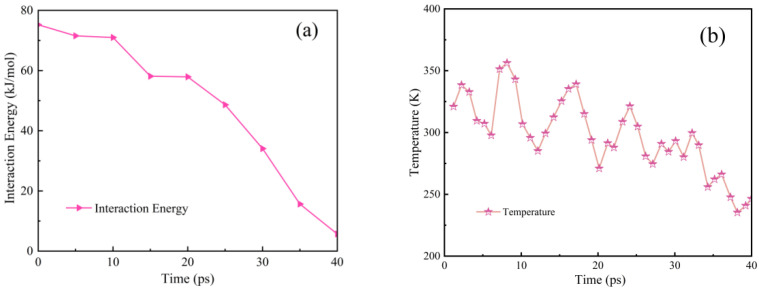
Interfacial interaction energy and matrix temperature during GNS pull-out process in 2%GNS/PEEK/PTFE: (**a**) interfacial interaction energy, (**b**) temperature of the composite systems.

To gain a deeper understanding of the atomic-scale origins of these mechanical property enhancements, the interfacial interactions between GNSs and the PEEK/PTFE composites and temperature were further investigated through pull-out simulations, as shown in Figure 8a,b. The energy profiles reveal that as the GNS was pulled out, the interfacial energy decreased from an initial value of 74 kJ/mol to 5.5 kJ/mol. This change indicates strong interfacial interactions (van der Waals and electrostatic forces) between GNSs and the matrix. Initially, the GNS adsorbs numerous PEEK/PTFE molecular chains through these interactions. As pull-out process progressed, the contact area between the GNS and the matrix decreased, reducing the number of adsorbed molecular chains and consequently lowering the interfacial energy. A high interfacial binding energy implies strong bonding between the GNS and the matrix, enabling effective load transfer under tension and compression and preventing interfacial debonding. This strong interfacial bonding constitutes the fundamental mechanism by which the GNS enhances the mechanical properties of the composite.

During the GNS pull-out process, the matrix temperature gradually dropped from 298 K to 285 K. The underlying mechanism of the temperature reduction involves the matrix consuming energy to overcome the interfacial interactions between GNS and molecular chains. This energy consumption lowers the total energy of the system, leading to a temperature drop. This phenomenon further confirms the presence of strong GNS matrix interactions, which demand external energy to disrupt. Macroscopically, this means that a higher load must be applied to induce material failure, thereby enhancing tensile strength and reducing compressive deformation.

In summary, the reinforcement mechanism of GNSs involves strong interfacial adsorption that enables effective load transfer, thereby curbing matrix deformation and failure. Moreover, the composite with 2 wt% GNS loading, benefiting from its uniform dispersion of GNSs, exhibits the strongest interfacial interactions and thus achieves the most effective reinforcing effect.

### 3.4. Tribological Properties of Composites: MD Simulation Results and the Enhancement Mechanism of GNS

The friction coefficient and wear rate of the composites were calculated via MD simulations, with the corresponding data summarized in Table 2. This approach was designed to quantitatively elucidate the regulatory effects of GNSs on the tribological performance of the composites at the atomic scale, as well as to validate the reliability of the macroscopic experimental results.

As shown in Table 2, the simulated friction coefficient of the PEEK/PTFE composite incorporating 2 wt% GNS is 0.1227, which is 19.59% higher than that of the matrix. Notably, this increment magnitude is lower than those of the composites modified with 1 wt% and 3 wt% GNS. Meanwhile, it exhibits a simulated wear rate reduction of 21.24% relative to the matrix, with this reduction amplitude being significantly greater than that of the composites containing 1 wt% and 3 wt% GNS. From the perspective of friction behavior regulation, the introduction of 2 wt% GNS enables the formation of a homogeneous “sliding-support” layer at the friction interface. This unique interfacial structure not only mitigates the adhesion of molecular chains but also minimizes the fluctuation of frictional resistance induced by GNS agglomeration, thereby yielding the minimal increment in friction coefficient. In terms of wear rate, the incorporation of 2 wt% GNS can effectively inhibit the ploughing action of the iron counterface on the matrix and mitigate the detachment of matrix atoms, thereby minimizing the wear rate of the composite. By contrast, a GNS loading of 1 wt% only affords partial protection against wear, whereas a higher loading of 3 wt% tends to induce GNS agglomeration, which in turn exacerbates localized wear of the composite.

The simulation results confirm that the incorporation of 2 wt% GNS achieves the optimal enhancement effect on the wear resistance of PEEK/PTFE composites at the atomic scale, and this conclusion is in good agreement with the macroscopic experimental data. It should be emphasized that MD simulations are intended to qualitatively interpret the variation trend of tribological properties, rather than to achieve quantitative matching of specific values. The quantitative discrepancies mainly originate from four aspects. First, the sliding velocity adopted in the simulations is much higher, which overestimates the increment in friction coefficient. Secondly, the wear rate in simulations is calculated based on the proportion of interfacial atomic detachment, whereas the wear rate in experiments is derived from the quantified mass loss of specimens. This leads to an essential difference in the response mechanisms of wear rate. Thirdly, the thermostatting strategy in simulations does not cover the friction interface, which induces interfacial heating and further amplifies the deviation of friction coefficient. Finally, the simplified smooth contact model adopted in simulations neglects the micro-roughness of actual specimen surfaces, ultimately resulting in inconsistent quantitative results.

**Figure 9:**
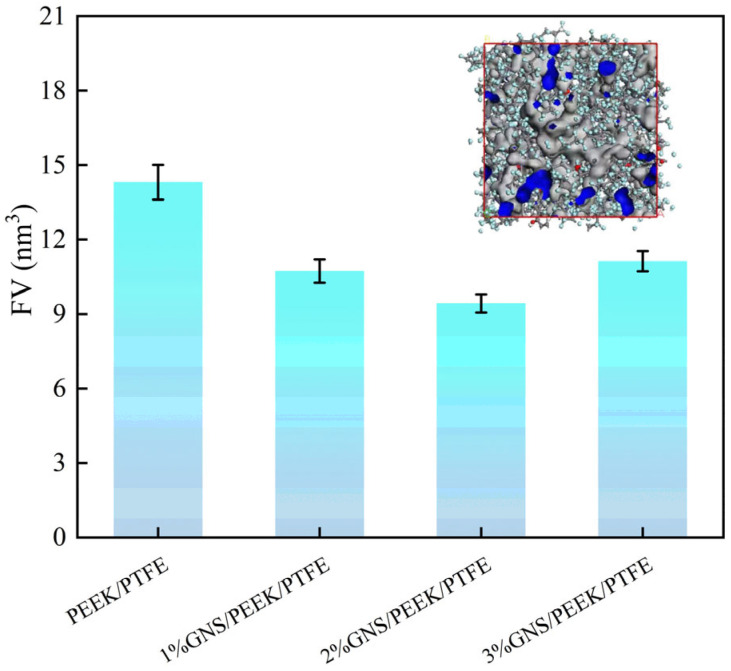
Free Volume of GNS/10%PEEK/PTFE composites with different GNS contents.

To elucidate the regulatory effect of the GNS on the PTFE/PEEK composites, the FV of four composites was calculated and the results are illustrated in Figure 9. FV serves as the space available for the movement of molecular chains. Accordingly, a smaller FV value denotes more constrained molecular chain mobility and a more compact material microstructure. MD simulation results demonstrated that the FV values of the composites with 1 wt%, 2 wt%, and 3 wt% GNS loading were 10.73 nm^3^, 9.43 nm^3^, and 11.13 nm^3^, respectively. Compared to the matrix (14.31 nm^3^), these values correspond to reductions of 25.01%, 34.10%, and 22.22%, respectively. Notably, the composite with 2 wt% GNS exhibited the minimum FV, which can be ascribed to the homogeneous dispersion of GNSs at this optimal dosage. The layered structure of the GNS fills the gaps between the molecular chains of the matrix and restricts their free motion via interfacial adsorption. The consequent enhancement in structural compactness is conducive to mitigating shear-induced molecular chain scission during the friction process, thereby contributing to a reduced wear rate. This finding is in agreement with the macroscopically observed low wear rate of the 2 wt% GNS composite.

**Figure 10:**
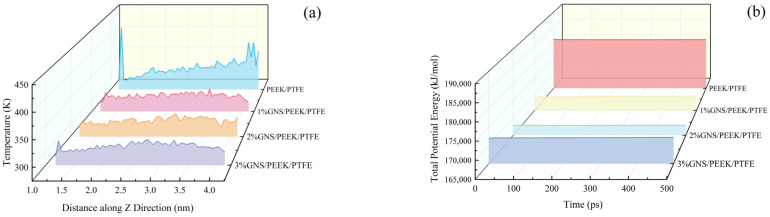
Variations in temperature and total potential energy of GNS/10%PEEK/PTFE composites during friction: (**a**) temperature of the friction interface, (**b**) total potential energy of the composite systems.

To further investigate the influence of GNSs on the tribological properties of PEEK/PTFE composites, the temperature of the friction interface in the GNS/PEEK/PTFE composites during friction was analyzed, as shown in Figure 10a. Friction interface temperature directly reflects energy dissipation during sliding: a lower temperature indicates less energy dissipation and less severe wear. The simulation results demonstrated that the peak temperatures of the composites with 1 wt%, 2 wt%, and 3 wt% GNS loading reached 315.45 K, 305.93 K, and 321.46 K, respectively. Relative to the PEEK/PTFE matrix (416.30 K), the aforementioned values correspond to temperature reductions of 24.22%, 25.89%, and 22.78%, respectively. Furthermore, the composite incorporating 2 wt% GNS exhibited an average temperature of 307.67 K, which was 5.45% lower than that of the PEEK/PTFE matrix (325.46 K). This phenomenon can be ascribed to the fact that 2 wt% GNS loading enables efficient dispersion of frictional stress and mitigation of local energy concentration. Additionally, GNSs can dissipate a fraction of frictional energy via interlayer sliding, thereby contributing to the reduction in interfacial temperature. In contrast, insufficient dispersion of 1 wt% GNS and severe agglomeration of 3 wt% GNS tend to induce pronounced energy concentration, which consequently results in a less significant temperature reduction effect. The reduced interfacial temperature during the friction process effectively suppresses thermal softening of the polymer matrix and enhances resistance to adhesive wear. This temperature-dependent effect accounts for the minimal wear rate exhibited by the composite with 2 wt% GNS loading.

In addition to temperature variations, changes in the total potential energy of the composite system also reflect the deformation and motion states of molecular chains from an energetic perspective. Specifically, lower potential energy corresponds to more stable molecular chain motion and higher resistance to shear deformation. To further elucidate the regulatory mechanism of GNSs on the tribological properties of PEEK/PTFE composites based on energy evolution, the total potential energy of the composites was analyzed, as shown in Figure 10b. During the 0–500 ps friction process, the average total potential energies of the composites with 1 wt%, 2 wt%, and 3 wt% GNS loading were 169,243 kJ/mol, 167,864 kJ/mol, and 172,015 kJ/mol, respectively. These values represent reductions of 6.47%, 7.22%, and 4.93% compared with the matrix (180,941 kJ/mol). Notably, the composite with 2 wt% GNS loading exhibited the lowest potential energy, indicating that its molecular chains underwent the minimal degree of deformation and motion under frictional shear. This finding is consistent with the minimum FV observed. The GNS restricts the motion space of molecular chains and reduces shear deformation, thereby contributing to the enhanced wear resistance of the composites.

## 4. Conclusions

This study elucidates the regulatory effect of GNS content on the microstructure, mechanical properties, and tribological behaviors of PTFE/PEEK composites through multi-scale characterization and analysis.

2 wt% is identified as the optimal GNS loading, which synergistically optimizes the mechanical and tribological performance of the composite. Compared with the matrix, the composite with 2 wt% GNS exhibits a 10.58% increase in tensile strength and an 11.07% reduction in compressive deformation. Concomitantly, it achieves a 17.88% decrease in the wear rate with only a marginal 2.51% increment in the friction coefficient. This formulation achieves an excellent balance between low friction and high wear resistance.

MD simulations confirm that 2 wt% GNS can be uniformly dispersed in the composite, fill the molecular gaps, and reduce the FV by 34.10%. The GNS restricts the movement of molecular chains, enhances interfacial bonding, and improves the structural compactness of the composite. It improves mechanical properties mainly by stress transfer, rigid network construction, and plastic deformation inhibition. Meanwhile, it can disperse frictional stress, suppress thermal softening, alleviate ploughing effects, reduce wear debris generation, and ultimately achieve the enhancement of wear resistance.

When the mass fraction of graphene is 1%, the insufficient loading results in poor filling efficiency, thus failing to achieve the reinforcing effect. In contrast, at a mass fraction of 3%, graphene nanosheets (GNSs) tend to agglomerate severely, which gives rise to structural defects, impedes interfacial stress transfer, and ultimately leads to the inferior comprehensive performance of the composites.

The 2 wt% GNS/PEEK/PTFE composite fabricated in this study exhibits excellent mechanical strength and wear resistance, thus demonstrating broad application prospects under high-pressure and oil-free lubrication conditions. It is particularly suitable for manufacturing critical components such as self-lubricating bearings, sliding seals, and piston rings used in aerospace engines and automotive powertrain systems. Nevertheless, this study still has certain limitations. Amorphous models were adopted for both PTFE and PEEK matrices during the modeling process, without considering the potential influence of crystalline structures on the micro-mechanisms and macroscopic properties of the composite. Moving forward, future research will focus on constructing a PTFE/PEEK crystalline-amorphous hybrid matrix model to systematically investigate the regulatory effects of the crystallinity, crystal orientation, and crystalline region distribution on mechanical properties, and tribological behaviors of GNS/PEEK/PTFE composites. Meanwhile, long-term durability tests under actual service conditions will be conducted to verify the reliability of the composite in engineering applications.

## Figures and Tables

**Table 1:** Composition of the PTFE composites (wt%).

Composites	PTFE	PEEK	GNS
PEEK/PTFE	90	10	0
1% GNS/PEEK/PTFE	89.1	9.9	1
2% GNS/PEEK/PTFE	88.2	9.8	2
3% GNS/PEEK/PTFE	87.3	9.7	3

**Table 2:** Coefficient of friction (COF) and Attrition rate (AR) of GNS/10%PEEK/PTFE composites.

GNS Blend Ratio	COF	Increase Percentage (%)	AR (%)	Decrease Percentage (%)
0%	0.1026	0	37.34	0
1%	0.1431	36.26	34.38	7.92
2%	0.1227	19.59	29.41	21.24
3%	0.1244	21.24	34.92	6.48

## Data Availability

The raw data supporting the conclusions of this article will be made available by the authors on request.
